# Orbiviruses in Rusa Deer, Mauritius, 2007

**DOI:** 10.3201/eid1702.101293

**Published:** 2011-02

**Authors:** Ferran Jori, Matthieu Roger, Thierry Baldet, Jean-Claude Delécolle, Jacqueline Sauzier, Mahmad Reshad Jaumally, François Roger

**Affiliations:** Author affiliations: French Agricultural Research Center for International Development (CIRAD), Montpellier, France (F. Jori, F. Roger);; University of Pretoria, Pretoria, South Africa (F. Jori);; CIRAD, Sainte-Clotilde, La Réunion Island, France (M. Roger);; CIRAD, Cotonou, Bénin (T. Baldet);; Université de Strasbourg, Strasbourg, France (J.C. Delécolle);; Mauritius Deer Farming Coopérative Society Ltd, Curepipe, Mauritius (J. Sauzier);; Ministry of Agro Industry and Fisheries, Réduit, Mauritius (M.R. Jaumally)

**Keywords:** Rusa deer, bluetongue, epizootic hemorrhagic disease, viruses, orbivirus, Culicoides, midges, Mauritius, letter

**To the Editor:** Bluetongue and epizootic hemorrhagic disease are caused by orbiviruses transmitted by *Culicoides* spp. biting midges (Diptera: Ceratopogonidae). These diseases are restricted to regions where their vectors exist (*1*) and seem to be expanding to previously unaffected areas (*2*). Infection of wild and domestic ruminants is common. Bluetongue virus (BTV) causes severe clinical disease in certain breeds of sheep; BTV and epizootic hemorrhagic disease virus (EHDV) cause clinical disease in some species of deer (*3*,*4*).

Rusa deer (*Cervus timorensis rusa*), originally from Indonesia, are found in diverse countries in the Pacific region (Papua New Guinea, New Caledonia, New Zealand, and Australia). Introduced to the island of Mauritius in 1639, they are commonly raised in high numbers (≈60,000) for meat production (*5*). Mauritius is considered free from major livestock diseases; its animal health surveillance is based mainly on clinical monitoring and inspection of carcasses at slaughter. To our knowledge, circulation of orbiviruses in Rusa deer has not been reported in detail in any country where this deer is present.

Our study was an initial screening survey of the deer population on the island. A total of 369 deer, representing 28 private farms, were chosen from a list of 42,959 deer. Blood was collected at slaughter, and serum samples were sent to Onderstepoort Veterinary Institute, South Africa, to be tested for antibodies against orbiviruses with a homemade indirect ELISA. To distinguish between BTV and EHDV, samples positive by indirect ELISA were tested for BTV antibodies with the competitive ELISA produced by the Institute for Animal Health (Pirbright, UK). Of the samples positive by competitive ELISA, 3 were tested by serum neutralization against the 24 BTV serotypes (cutoff value >16).

Simultaneously, *Culicoides* spp. midges were trapped in Onderstepoort-type blacklight traps at 3 deer farms in coastal areas (Figure). Trapping was conducted 1 night at each farm, during optimal weather conditions. Midges were kept at room temperature in 95% ethanol until sent to Strasbourg University, France, for identification.

**Figure:**
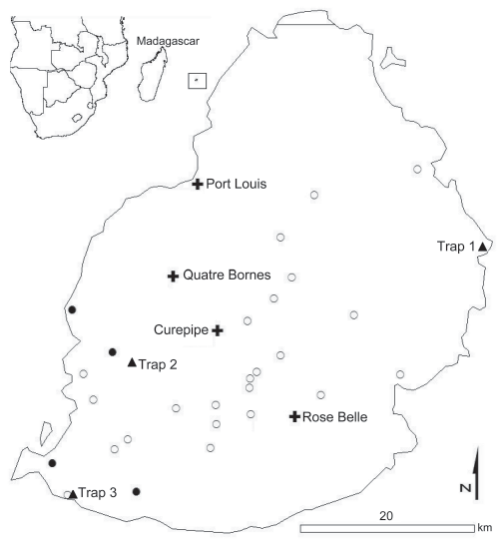
Location of farms where Rusa deer were sampled (open circles), herds with orbivirus-seropositive deer (closed circles), biting midge collection sites (triangles), and main cities (crosses) in Mauritius. Most (99%) *Culicoides* spp. midges were trapped at sites 1 and 3. Inset show location of Mauritius (in square) in relation to Africa and Madagascar.

Of the 369 deer serum samples tested, 15 were positive for BTV and 5 for EHDV; seroprevalence was 4.1% for BTV (95% confidence interval 2.0%–6.1%) and 1.3% for EHDV (95% confidence interval 2.0%–6.1%). No significant differences were observed for sex (χ^2^ = 0.05, p = 0.82). Antibodies (indirect ELISA) against orbiviruses were more prevalent among adults (χ^2^ = 4.56, p = 0.03). The 3 samples tested by serum neutralization had high titers against BTV-2 (256, 256, and 128) and lower but positive titers against BTV-17 (32 in each), BTV-10 (16 in each), and BTV 21 (32 in 1). Despite reports of clinical signs in other deer species infected with both viruses (*3*,*4*), no signs of bluetongue or epizootic hemorrhagic disease were reported for the Rusa deer population in our survey. This absence of clinical disease might be the result of natural resistance of this species to orbiviruses or to the fact that the circulating serotypes are endemic to the area.

A total of 13,356 *Culicoides* spp. midges were obtained; 12% were identified as C. *imicola* (1,459 females, 138 males) and 88% as *C. enderleini* (8,800 females, 2,878 males). The former species has been reported in Mauritius (*6*). In our study, the positive serum came from deer on 4 farms located in the coastal area. This finding could indicate a higher abundance and activity of *C. imicola* midges in coastal areas, where climate and altitude are more favorable for the vector.

Serum neutralization results suggested that at least 4 serotypes could have been circulating in deer from Mauritius. During outbreaks of both viruses in neighboring Réunion Island, several serotypes of BTV were isolated from sheep (*7*,*8*) and of EHDV from cattle (*9*). However, BTV serotypes 17 and 21 have never been isolated from Indian Ocean countries, and serotype 21 has been detected only in Australia (*2*). Equally, diverse BTV serotypes circulate at different locations on the Indian Ocean and the east coast of Africa (*2*,*5*,*6*).

Our results provide serologic indication that EHDV and BTV circulate in Rusa deer in Mauritius. The large population of Rusa deer can represent a potential reservoir host for those viruses and a risk for transmission to other ruminants in Mauritius and neighboring countries. However, Rusa deer could be used as a sentinel population to regularly monitor the circulation of orbiviruses and the introduction of new serotypes to Mauritius. To detect and isolate circulating serotypes and genotypes of these viruses in ruminant species and in potential vectors in Mauritius, further research is needed. In addition, the extent of both viruses and the distribution of *Culicoides* midges over the island should be investigated in more detail.

## References

[1] Mellor PS, Boorman J, Baylis M. *Culicoides* biting midges: their role as arbovirus vectors. Annu Rev Entomol. 2000;45:307–40. 10.1146/annurev.ento.45.1.307 10761580

[2] MacLachlan NJ, Guthrie AJ. Re-emergence of bluetongue, African horse sickness, and other orbivirus diseases. Vet Res. 2010;41:35. 10.1051/vetres/2010007 20167199 PMC2826768

[3] Haigh JC, Mackintosh C, Griffin F. Viral, parasitic and prion diseases of farmed deer and bison. Rev Sci Tech. 2002;21:219–48. 11974612 10.20506/rst.21.2.1331

[4] Gaidos JK, Crum JM, Davidson WR, Cross SA, Owen SF, Stallknecht DE. Epizootiology of an epizootic hemorrhagic disease outbreak in West Virginia. J Wildl Dis. 2004;40:383–93. 15465704 10.7589/0090-3558-40.3.383

[5] Chardonnet P, Des Clers B, Fischer J, Jori F, Lamarque F. The value of wildlife. Rev Sci Tech. 2002;21:4–47. 11974626 10.20506/rst.21.1.1323

[6] Boorman J, Mellor PS. *Culicoides* vectors of bluetongue and African horse sickness viruses in Mauritius. Med Vet Entomol. 1992;6:306. 10.1111/j.1365-2915.1992.tb00622.x 1330088

[7] Breard E, Sailleau C, Hamblin C, Zientara S. Bluetongue virus in the French island of Réunion. Vet Microbiol. 2005;106:157–65. 10.1016/j.vetmic.2004.11.018 15778021

[8] Barré N, Eramus BJ, Gautier A, Reme A, Valin R. La bluetongue, nouvelle maladie des ovins à la Réunion (Océan Indien). Rev Elev Med Vet Pays Trop. 1985;38:16–21. 3018871

[9] Breard E, Sailleau C, Hamblin C, Graham SD, Gourreau JM, Zientara S. Outbreak of epizootic haemorrhagic disease on the island of Réunion. Vet Rec. 2004;155:422–3. 10.1136/vr.155.14.422 15508843

